# Brain Connectivity Based Prediction of Alzheimer’s Disease in Patients With Mild Cognitive Impairment Based on Multi-Modal Images

**DOI:** 10.3389/fnhum.2019.00399

**Published:** 2019-11-15

**Authors:** Weihao Zheng, Zhijun Yao, Yongchao Li, Yi Zhang, Bin Hu, Dan Wu, for the Alzheimer’s Disease Neuroimaging Initiative

**Affiliations:** ^1^Key Laboratory for Biomedical Engineering of Ministry of Education, College of Biomedical Engineering and Instrument Sciences, Zhejiang University, Hangzhou, China; ^2^School of Information Science & Engineering, Lanzhou University, Lanzhou, China; ^3^Department of Neurology, The First Affiliated Hospital, School of Medicine, Zhejiang University, Hangzhou, China

**Keywords:** Alzheimer’s disease (AD), mild cognitive impairment (MCI), multi-modal connectivity, early diagnosis, individual network

## Abstract

Structural and metabolic connectivity are advanced features that facilitate the diagnosis of patients with Alzheimer’s disease (AD) and mild cognitive impairment (MCI). Connectivity from a single imaging modality, however, did not show evident discriminative value in predicting MCI-to-AD conversion, possibly because the inter-modal information was not considered when quantifying the relationship between brain regions. Here we introduce a novel approach that extracts connectivity based on both structural and metabolic information to improve AD early diagnosis. Principal component analysis was performed on each imaging modality to extract the key discriminative patterns of each brain region in an independent auxiliary domain composed of AD and normal control (NC) subjects, which were then used to project the two subtypes of MCI to the low-dimensional space. The connectivity between each target brain region and all other regions was quantified via a multi-task regression model using the projected data. The prediction performance was evaluated in 75 stable MCI (sMCI) patients and 51 progressive MCI (pMCI) patients who converted to AD within 3 years. We achieved 79.37% accuracy, with 74.51% sensitivity and 82.67% specificity, in predicting MCI-to-AD progression, superior to other existing algorithms using either structural and metabolic connectivities alone or a combination thereof. Our results demonstrate the effectiveness of multi-modal connectivity, serving as robust biomarker for early AD diagnosis.

## Introduction

Alzheimer’s disease (AD) is the most common neurodegenerative disease characterized by short-term memory loss and a decline of cognitive functions, including executive, visuospatial abilities, and language (Braa and Braak, 1991). Mild cognitive impairment (MCI) is referred to as the prodromal stage of AD, which is accompanied by a measurable impairment in memory, without the loss of general cognitive functioning (Petersen et al., 2001; Morris and Cummings, 2005). Currently, over 50 million people have been diagnosed with AD worldwide, and this number will be nearly double by 2030 (Patterson, 2018). Moreover, as the intermedia stage between normal control (NC) and AD, over one-third of patients with MCI, on average, will progress to AD within 5 years (Ward et al., 2013). Because AD is an incurable disease that has become a serious global issue, developing diagnostic biomarkers for early prediction of the conversion from MCI to AD is of great importance, which, however, remains a challenge.

Brain network analysis is an efficient tool in characterizing topological organization of the brain (e.g., functional integration and segregation), which has been widely used in investigating cerebral abnormalities caused by mental disorders, such as AD and MCI (Tijms et al., 2013; Kong et al., 2015; Kim et al., 2016; Wang et al., 2016). However, unlike a functional network that can be built based on the image of a single subject, the construction of morphological and metabolic networks are mostly at the group-level, which cannot be used to represent individual properties for diagnostic purposes (He et al., 2008; Yao et al., 2010, 2015; Evans, 2013). To address this issue, several methods for extracting brain networks from individual magnetic resonance (MRI) and positron emission tomography (PET) images have been proposed. For example, at the brain parcel level, Pearson correlation, Euclidean distance, mutual information, and the Kullback–Leibler divergence of probability density function were, respectively, used to measure the relationship between the properties (e.g., cortical thickness, volume and metabolism) of distinct brain regions (Wee et al., 2013; Kong et al., 2014; Raamana et al., 2015; Zheng et al., 2015; Jiang et al., 2017; Li et al., 2017; Liu et al., 2017); at the voxel-level, Pearson correlations between gray matter density of small patches consisting of a serial of voxels (e.g., 3 × 3 × 3 voxels in each patch) were used to construct the covariance matrix (Tijms et al., 2012). These networks were reported with “small-world” organization and altered network matrices with the progression of AD (Tijms et al., 2013; Kong et al., 2015; Kim et al., 2016; Wang et al., 2016); and achieved an evident performance superior to original anatomical and metabolic features in classifying AD and MCI cohorts from the NCs (Wee et al., 2013; Liu M. et al., 2014; Zheng et al., 2015; Yao et al., 2016; Zhao et al., 2017).

Though informative, these networks are based on paired relationships, which omit the nature of inter-play in the brain (one brain region has “first-order” connections with only a few of the other regions) (Sporns et al., 2000) that may contain crucial information for disease diagnosis. Recent studies applied the sparse regression model (e.g., LASSO) to functional MRI to quantify such interaction patterns and established the “hyper-functional network,” which was powerful in diagnosing patients with MCI, attention deficit hyperactivity disorder (ADHD) and major depressive disorder (MDD) (Ryali et al., 2012; Jie et al., 2014, 2016; Guo et al., 2018; Li et al., 2019). We further extended this connectivity extraction strategy to construct a cortical network based on multiple morphological features in the brain (so called multi-feature based network, MFN) (Zheng et al., 2018). The connectivity of the MFN achieved outstanding performance that is superior to both anatomical features and some of the aforementioned structural networks in identifying patients with AD, MCI, and autism spectrum disorder (ASD) (Zheng et al., 2018, 2019), suggesting that cortico-cortical structural connectivity may possess critical information for AD/MCI diagnosis. However, the features of the MFN failed to classify the MCI convertors from non-convertors (Zheng et al., 2018), possibly due to the subtle differences between the two cohorts that cannot be captured by networks that only contain morphological information. A recent study indicated that FDG-PET and T1 images may characterize the features of AD from different perspectives, e.g., hypometabolism is more related to the pathological processes and clinical severity of AD, whereas cortical atrophy is more related to the cognitive reserve (Benvenutto et al., 2018). We thus speculated that the cortico-cortical connectivity that combines both metabolic and morphological information may further enhance the prediction performance of MCI-to-AD conversion, because this connectivity synergistically depicts the abnormal changes on both sides.

In the present study, we aimed to develop a brain connectivity that jointly reflects the high-order morphological and metabolic interactions to improve the prediction accuracy of MCI conversion. A framework to construct brain networks based on multi-modal images (MRI and PET) was proposed. For each imaging modality, we trained the principal patterns of each brain region with large variances in categorization using an independent auxiliary dataset consisting of AD and NC subjects. The connectivity extraction of each imaging modality in the target dataset was then treated as a single task, and a multi-task sparse regression model (Nie et al., 2010) with *l*_1_/*l*_2_-norm regularization was utilized to quantify the connectivity by jointly identifying brain regions that have both robust structural and metabolic associations with the target region. We examined the diagnostic performance of the multi-modal connectivity (MMC) by cross-validating the results with a support vector machine (SVM) (Vapnik, 2000). The diagnostic performances of the MMC was compared to single-modal connectivities, as well as other existing connectivity analysis methods, based on MRI and PET images.

## Materials and Methods

### Subjects

Images were obtained from the Alzheimer’s Disease Neuroimaging Initiative (ADNI) database^1^. Subjects had both MRI and ^18^F-fluorodeoxyglucose PET (FDG-PET) images were included. Notably, we selected MCI patients who have at least 3-year follow up information from the baseline. Finally, baseline images of 75 NCs, 78 patients with AD, 75 stable MCI (sMCI) patients, and 51 progressive MCI (pMCI) patients were included. The general diagnostic criteria were defined in the ADNI protocol. Briefly, the NCs were scored between 24 and 30 (inclusive) on the Mini-Mental State Examination (MMSE) (Folstein et al., 1975) and had a Clinical Dementia Rate (CDR) (Morris, 1993) of 0, was non-depressed and non-demented. The MCI group were scored between 24 and 30 (inclusive) on the MMSE, had a CDR of 0.5, with memory complaints and objective memory loss, but no significant levels of impairment in other cognitive domains, and no presence of dementia. In the present study, subjects who progressed to AD within 3 years from baseline were defined as pMCI, and subjects who did not convert to AD within the same time period were define as sMCI patients. The patients with AD were scored between 20 and 26 (inclusive) on the MMSE and had a CDR of 0.5 or 1, and met NINCDS/ADRDA criteria (McKhann et al., 1984) for probable AD. Table 1 summarizes the characteristics of the four cohorts. No significant difference was found between MRI non-convertors and convertors in age [*t*_(124)_ = 1.0473, *p* = 0.2987] and gender [χ^2^_(1)_ = 0.2054, *p* = 0.6504].

**TABLE 1:** Demographic information of participants.

**Cohorts**	**Auxiliary domain**		**Target domain**	
	**AD**	**NC**	**sMCI**	**pMCI**
Age	75.96 ± 7.24	76.26 ± 5.16	76.22 ± 6.28	74.95 ± 7.29
Sex (M/F)	46/32	45/30	50/25	32/19
MMSE	23.40 ± 2.15	28.91 ± 1.19	27.41 ± 1.62	26.80 ± 1.69
CDR	0.5 or 1	0	0.5	0.5

*M/F, male/female; MMSE, Mini-Mental State Examination (MMSE); CDR, Clinical Dementia Rate.*

### Imaging Data

Structural images we downloaded were baseline T1 weighted MRI acquired from 1.5T scanners. All the images were controlled for quality and underwent corrections for geometry distortion and intensity non-uniformity^2^. FDG-PET images were acquired 30 to 60 min post-injection and reviewed for quality at the University of Michigan. All PET images were co-registered, averaged, reoriented, interpolated into standard resolution (160 × 160 × 96 voxels, 1.5 mm^3^ voxel size), and normalized for intensity^3^.

## Methods

### Preprocessing

MRI and PET images were preprocessed using Statistical Parametric Mapping (SPM12) software. The preprocessing of MR images was conducted using the CAT12 toolbox^4^ with the default setting. Briefly, the process started with registration using affine, followed by realignment, bias correction for inhomogeneity, and the segmentation of gray matter (GM), white matter (WM) and cerebral spinal fluid (CSF) (Ashburner and Friston, 2005). Then GM segmentations were spatially normalized to a prior template in the MNI152 space using the DARTEL (Diffeomorphic Anatomical Registration using Exponential Lie Algebra) algorithm (Ashburner, 2007), and the spatial resolution of images were resampled to 2 × 2 × 2 mm. Normalized images were corrected for non-linear deformation of the spatial normalization to generate modulated normalized images, which were then smoothed using a 5-mm full width at half maximum (FWHM) Gaussian kernel. For the preprocessing of PET images, each image was co-registered to the MRI of the same subject and then normalized using the deformation field of the corresponding MRI. The normalized images were smoothed using a 5-mm FWHM Gaussian kernel. The cerebral part of the two image modalities were segmented into hundreds of brain regions by registering a parcellation atlas to the template image in the MNI152 space. Here, we used Human Brainnetome Atlas for brain parcellation, which is a voxel-based parcellation containing 246 brain regions that builds upon multi-modal connectivity information of 40 healthy adults from the Human Connectome Project (HCP) database (Fan et al., 2016).

### Overview of Multi-Modal Network

Human brain is a highly interactive system, in which a connectivity may link multiple high-related brain regions rather than only two of them. Such connective patterns were found in both functional and structural brain networks (Ryali et al., 2012; Zheng et al., 2018). In the present study, we took a further step to extract networks based on multiple imaging modalities to facilitate the diagnosis of MCI-to-AD conversion. The multi-modal network is denoted as *G* = (*V*,*E*), with a node set *V* and an edge set *E*. Here, *V* is a set of brain regions and *E* is consisted by the MMC.

**FIGURE 1:**
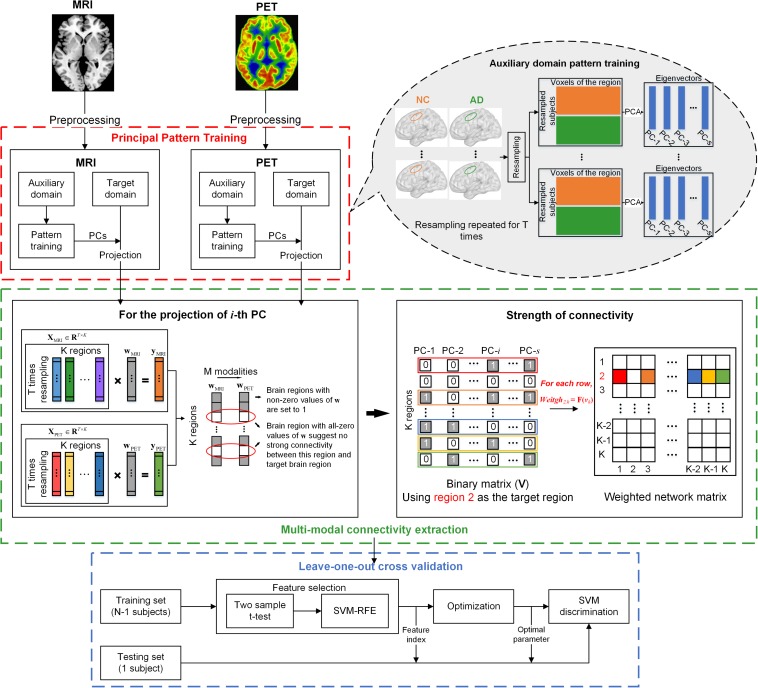
Schematic representation of the MMC extraction and the classification process. Principal pattern training (red box): for each modality, PCA was applied to each brain region of the auxiliary domain data consisted by AD and NC subjects. The dataset used for PCA was derived from the bagging process, which randomly picked subjects with a replacement to form a new sample set. This process was repeated for *T* times, and *T* matrices of eigenvectors were obtained for each brain region. Multi-modal connectivity extraction (green box): For each modality, we projected each brain region of observations in the target domain (sMCI and pMCI) to a low dimensional feature space using the eigenvectors of the corresponding region. For each brain, suppose the *i*-th principal component (eigenvector) was used for projection, then a projected vector of *T* × 1 could be obtained. The projected vectors of all brain regions using the *i*-th principal component (PC) of each region could form a data matrix **X**_*M*_ ∈ *R*^*T*×*K*^, where *K* is the number of brain regions, and *M* is the imaging modalities. The multi-task regression was performed with the vector of one brain region in **X**_*M*_, in turn, served as the target variable and the vectors of other regions as regressors. The non-zero regression coefficients were set to 1 to represent the connectivity between regressors and the target brain region. If *S* PCs were selected for projection, the aforementioned process was repeated *S* times. Then a binary vector of 1 × *S* could be obtained that represents the relationship between one regressor and the target brain region derived from *S* regression processes (as shown in the right of the green box). The binary vector was converted to decimal and normalized to represent the connectivity strength between the two brain regions. Cross validation: leave-one-out cross validation, with nested two-step feature selection and parameter optimization, was performed on MCI subjects (*N* = 75 pMCI + 51 pMCI) to examine the validity of the MMC.

The diagram illustrated in Figure 1 outlines the pipeline of the multi-modal network construction. Briefly, the MRI and PET images were first preprocessed and registered to a prior template. A principal component analysis (PCA) with a bagging strategy was then performed to each imaging modality in the auxiliary domain, consisted by AD and NC subjects, to extract the PCs of each brain region based on resampling the subjects with replacement. Regional data in the target domain, consisted by sMCI and pMCI subjects, were then projected to that feature space using the corresponding PCs of the brain region. The projected data were submitted to a multi-task sparse regression model to extract the connectivity between one target brain region and other regions. The technical details are provided below.

#### Bagging-Based Principal Component Analysis

In the present study, we applied PCA to the auxiliary domain to extract the principal patterns of the voxels in each brain region that have large variances between AD and NC. We speculated that the information possessed by the top PCs should also be able to contribute to discriminate AD-like (pMCI) and NC-like (sMCI) subjects. Studies have found that using classifiers trained by NC and AD subjects enhanced the performance of categorizing MCI convertors from non-convertors (Fan et al., 2008; Cheng et al., 2015), suggesting the effectiveness of using discriminative information trained by AD and NC subjects. Because the sample size of the neuroimaging dataset is usually small, the principal patterns that are estimated based on the small dataset may not be generalized across datasets. Therefore, we applied a bagging strategy to PCA, which trained the PCs of each brain region by random sampling the auxiliary samples with replacement for *T* times (*T* = 200 in this study) and ensuring the number of resampled subjects in each group was equal to the original dataset, to reduce the possible estimation bias of single time analysis (see the red box in Figure 1). Regional data of each subject in the target domain were then projected to a lower dimensional space using the PCs of each brain region. Finally, for each modality of a brain region, we obtained a projected data matrix with the dimension of *T* × *S*, where *S* is the number of PCs that were chosen for projection.

In the present study, we projected a brain region of an individual in the target domain using the *S* selected PCs of the same region. For example, we denoted the projected individual using the *i*-th PCs of each brain region as ${\text{X}}_{P\,C_{i}}^{M}=\left[x_{1}^{M},x_{2}^{M},\ldots ,x_{K}^{M}\right]\in {\text{R}}^{T\times K}$, with *M* imaging modalities, *K* brain regions and *T* times bootstrapping sampling. Therefore, if we selected *S* top rank PCs of each brain region for projection, the projected individual could be denoted as ${\text{I}}^{M}=\left\{{\text{X}}_{P\,C_{1}}^{M},{\text{X}}_{P\,C_{2}}^{M},\ldots ,{\text{X}}_{P\,C_{S}}^{M}\right\}$. This step not only extracts the regional principal patterns of the subjects in the target domain, but also expands the feature dimension in brain regions, which allows us to estimate inter-regional relationships on an individual level.

#### Multi-Modal Connectivity Extraction

The Multi-task sparse regression model was utilized instead of the paired correlation (e.g., Pearson correlation) and single-task model (e.g., LASSO), to quantify the relationships between a target brain region and multiple predictor regions (Yuan and Lin, 2006; Argyriou et al., 2007). As opposed to learning each task in isolation, multi-task learning exploits similarities across different learning tasks and can be used to jointly estimate the relationship between target variables and regressors across different types of tasks (Maurer et al., 2016), which therefore further takes intrinsic links of multimodal data into account.

For using each of the subset ${\text{X}}_{P\,C_{i}}^{M}\in {\text{R}}^{T\times K},\left(i\in S\right)$, the MMC among brain regions were generated by repeating the multi-task regression procedure *K* times, with each brain region in turn acting as the target variable and the remaining regions as the regressors. In the present study, we denoted each imaging modality as a single learning task, formulated as:

$$y_{P\,C_{i}}=X_{P\,C_{i}}^{m}w_{P\,C_{i}}^{m},\left(m\in M,i\in S\right)$$

where ${\text{X}}_{P\,C_{i}}^{m}$, ${\text{y}}_{P\,C_{i}}=x_{k}^{m},\left(k\in K\right)$, and ${\text{w}}_{P\,C_{i}}^{m}\in {\text{R}}^{K\times 1}$ were the regressor matrix, target vector, and regression coefficient, respectively. Notably, during the *k*-th regression, the regressor matrix ${\text{X}}_{P\,C_{i}}^{m}=\left[x_{1}^{m},\ldots ,x_{k-1}^{m},0,x_{k+1}^{m},\ldots ,x_{K}^{m}\right]\in {\text{R}}^{T\times K}$ contained all regional vectors, and $x_{k}^{m}$ was set to 0. The multi-task regression function was estimated *via l*_1_/*l*_*q*_-norm regularization (*q* = 2 in our study), which applies the *l*_1_ penalty over the regression coefficients that are derived from the *l*_2_ penalty for each input across tasks, thus allowing us to quantify the connections *via* jointly considering the information from two tasks (morphological and metabolic information). The *l*_1_/*l*_*q*_ penalty was formulated as follows:

$$\min_{\text{W}}\frac{1}{2}\sum_{m=1}^{M}||y_{P\,C_{i}}^{m}-X_{P\,C_{i}}^{m}w_{P\,C_{i}}^{m}|{|}_{2}^{2}+\lambda ||W_{P\,C_{i}}|{|}_{\ell_{1}/\ell_{q}}$$

where *λ* is the *l*_1_/*l*_*q*_-norm parameter specified as a ratio of the maximal sparse parameter whose value lies in the interval [0, 1]; ${\text{W}}_{P\,C_{i}}=\left[{\text{w}}_{P\,C_{i}}^{1},{\text{w}}_{P\,C_{i}}^{2},\ldots ,{\text{w}}_{P\,C_{i}}^{M}\right]\in {\text{R}}^{K\times M}$ is the combination of regression coefficients of multiple tasks, with each row representing the associations of the same brain region with target regions toward different tasks. The multi-task regression was conducted using the SLEP toolbox (Liu et al., 2009). We varied the value of λ in specified ranges (λ ∈ {1,2,…,10}×10^−3^) as suggested by Zheng et al. (2018), and evaluated the corresponding performance in terms of classification accuracy. The *W*_*PC_i*_ is used to measure the robustness of connectivity, in which rows with non-zero values suggest relatively strong relationships between the regressors and target regions, while rows with only zeros suggest a weak relationship. We merged the columns of **W**_*PC*_*i*__ into a binary vector with a *K*×1 dimension by setting the non-zero rows to 1 and all zero rows to 0. The pipeline of the aforementioned procedure is given in the left of the green box in Figure 1. Notably, because we selected *S* PCs for each brain region, this process was performed *S* times with ${\text{X}}_{P\,C_{i}}^{M}\left(i\in S\right)$ alternately serving as the model input. Therefore, for each target brain region, a binary matrix (**V** ∈ **R**^*K*×*S*^) was finally obtained that represents the relationships between this region and all other regions across multiple PCs.

MCI is known as a transition stage in AD, their alteration modes are similar (Braa and Braak, 1991; Karas et al., 2004; Rombouts et al., 2005) but vary in terms of connective strength (Yao et al., 2010; Binnewijzend et al., 2012). Therefore, the connective strength may possess critical information that should not be omitted in early diagnostic studies. However, because the regression coefficients of the back-ward model (e.g., multi-task regression model) cannot be used to represent the weights of features (Haufe et al., 2014), it limits the interpretation of connective strength between regressors and the target variable. To this end, we proposed to quantify the connective strength between two brain regions *via* a binary to decimal encoding strategy, defined as:

$$Weight_{j}=F\left(v_{k}\right)$$

$$=\frac{bin2dec\left(v_{k}\right)}{bin2dec\left(v\right)}$$

where **v**_*k*_ is the *k*-th row of binary matrix **V**; **v** is an all-one vector that has the same dimension of **v**_*k*_; *bin*2*dec* is a function to convert the binary sequence to a decimal number. We will take the right panel in the green box in Figure 1 as an example. For a binary matrix **V** derived from *S* multi-task regression models with region 2 as the target vector in each model, the *j*-th row of **V** represents the relationship between region 2 and region *j* across the *S* selected PCs. The connective weight between these two brain regions were then calculated *via* the equation above, which formed the *j*-th element of the second row of the final connective matrix. This method takes the information differences that the PCs possess into account and gives relatively large weights to the top-ranking PCs if they significantly contribute to the regression process. For each subject, the obtained network matrix was asymmetric with 246×(246−1) = 60,270 elements, which were concatenated to form a feature vector for feature selection. Notably, the asymmetric network does not reflect any communication mechanism or causality, rather, it represents region-to-region relationships.

### Feature Selection and Classification

We used the leave-one-out cross validation strategy to assess the classification performance of each feature type (see the blue box in Figure 1). The feature selection was applied to the training set of each validation process. Here, we used a two-step feature selection strategy to find a relative optimal feature subset for classification. In the first step, the two sample *t*-test was utilized to roughly filter-out the features that were less relevant to the discrimination, features with the top 20% *t* values were preserved. These features were then evaluated by a linear SVM-based recursive feature elimination (SVM-RFE) strategy (Guyon et al., 2002), which iteratively removes the features with the lowest discrimination performance. The ranking criterion of features was evaluated by the square term of weight coefficients (***w***^2^) derived from the SVM model, calculated as **w** = ∑_*k*_α_*k*_*y*_*k*_**x**_*k*_, where *y*_*k*_ and **x**_*k*_ is the class label and the feature vector, of sample *k*, respectively, α is an sparse index of support vectors. In each interaction, the 500 lowest ranking features were removed when the feature dimension was over 10,000; the step size was reduced to 50 for the last 10,000 features, 5 for the last 1,000 features, and 1 for the last 100 features (Zheng et al., 2019). The classification process was conducted using the LIBSVM toolbox (Chang and Lin, 2011). A nested five-fold cross validation was performed to optimize the parameter C of linear SVM in the range of 2*^β^*, β ∈ {−8,−7,…,8}. The classifier was then trained based on the selected features of the training set and the optimized parameter. To find the peak of accuracy, we increased the input features of the classifier from the top 10% of features derived from the first step of feature selection, with 5% increments of features. The accuracy, sensitivity, specificity, and area under the receiver operating characteristic (ROC) curve (AUC) were calculated for the performance assessment.

## Results

### Prediction Performance Using the MMC

**FIGURE 2:**
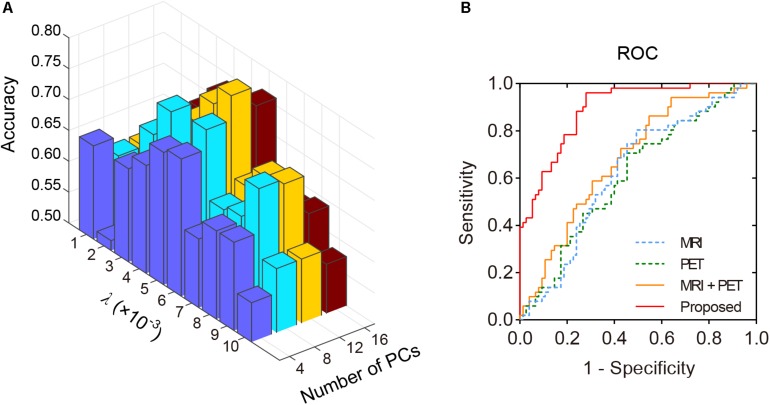
The classification performance of the MMC. **(A)** Classification accuracy with varying λ and number of selected PCs (*S*). The best accuracy was achieved at λ = 6×10^−3^ and *S=12*. **(B)** ROC curves at the best classification performance.

Structural and metabolic features showed limited discriminative power in identifying MCI convertors from non-convertors, which achieved an accuracy of 61.11 and 59.52%, respectively. The combination of these two types of features slightly improved the prediction accuracy to 62.70%, with AUC of 0.6790, but the classification performance remained limited. As shown in Figure 2A and Table 2, by varying *λ* and the number of selected PCs (*S*), the MMC achieved the best performance at λ = 6×10^−3^ and *S=12*, with an accuracy of 79.37%, accompanied by a high AUC of 0.8923 and small number of support vectors (76 on average). This is a significant improvement relative to the accuracy of the GM volume, metabolism and their combinations (χ^2^ test, *ps* < 0.005, Table 2). ROC curves of classification analyses using different feature types are shown in Figure 2B.

**TABLE 2:** Comparison of the classification performances based on the original imaging features and MMC.

**Features**	**Accuracy (%)**	**Sensitivity (%)**	**Specificity (%)**	**AUC**	***P* value**
GM volume	61.11	52.94	66.67	0.6235	0.0015
Metabolism	59.52	31.37	78.67	0.6110	0.0006
GM volume +	62.70	64.71	61.33	0.6790	0.0035
Metabolism					
MMC	79.37	74.51	82.67	0.8923	–

*MMC, multi-modal connectivity; AUC, area under curve; *P* value = compared the accuracy obtained by each feature in relative to the MMC by using χ^2^ test.*

### Connectivity With Significant Between-Group Difference

**FIGURE 3:**
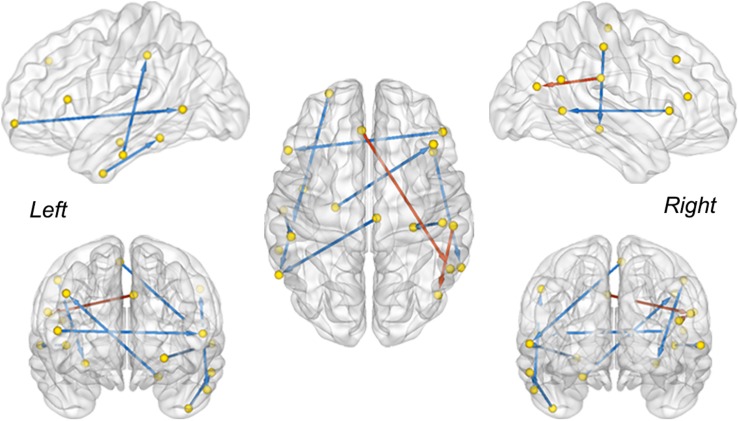
The MMC showing a significant difference between sMCI and pMCI. The red and blue color indicates increased and decreased strength of connectivity in the pMCI cohort, respectively (two sample *t*-test, FDR corrected, *q* < 0.05). The direction is used to differentiate the connections between the same-node pairs.

The between-group difference of MMC was evaluated *via* a two-sample *t*-test with a false discovery rate (FDR) correction (*q* < 0.05) (Benjamini and Hochberg, 1995). Connectivity with a significant difference between the MCI convertors and non-convertors is visualized in Figure 3. The MMC that connected the temporal lobe with the frontal and parietal cortices exhibited significant reductions, e.g., the connectivity between the left rostral hippocampus and right middle frontal gyrus (MFG, ventrolateral BA8), between the right middle temporal gyrus (MTG, dorsolateral BA37) and right inferior frontal gyrus (IFG, opercular BA44), between the left inferior temporal gyrus (ITG, intermediate lateral BA20) and left inferior parietal lobule (IPL, rostrodorsal BA40), and between the right postcentral gyrus (PoG) and right caudal hippocampus. In contrast, the connectivity that connected the right rostroventral IPL with left caudal IPL and left superior frontal gyrus (SFG, BA9) significantly increased. These results were in line with previous findings showing abnormal functional and structural alterations within these brain regions (e.g., the hippocampus and temporal cortex) (Baron et al., 2001; Frisoni et al., 2002; Matsuda et al., 2002; Chételat et al., 2008; Mosconi et al., 2009) and disrupted the connectivity related to them in patients with MCI and AD (Wang et al., 2007; Yao et al., 2010; Dai and He, 2014; Herholz et al., 2018).

### Comparisons With Other Connectivity Extraction Approaches

**FIGURE 4:**
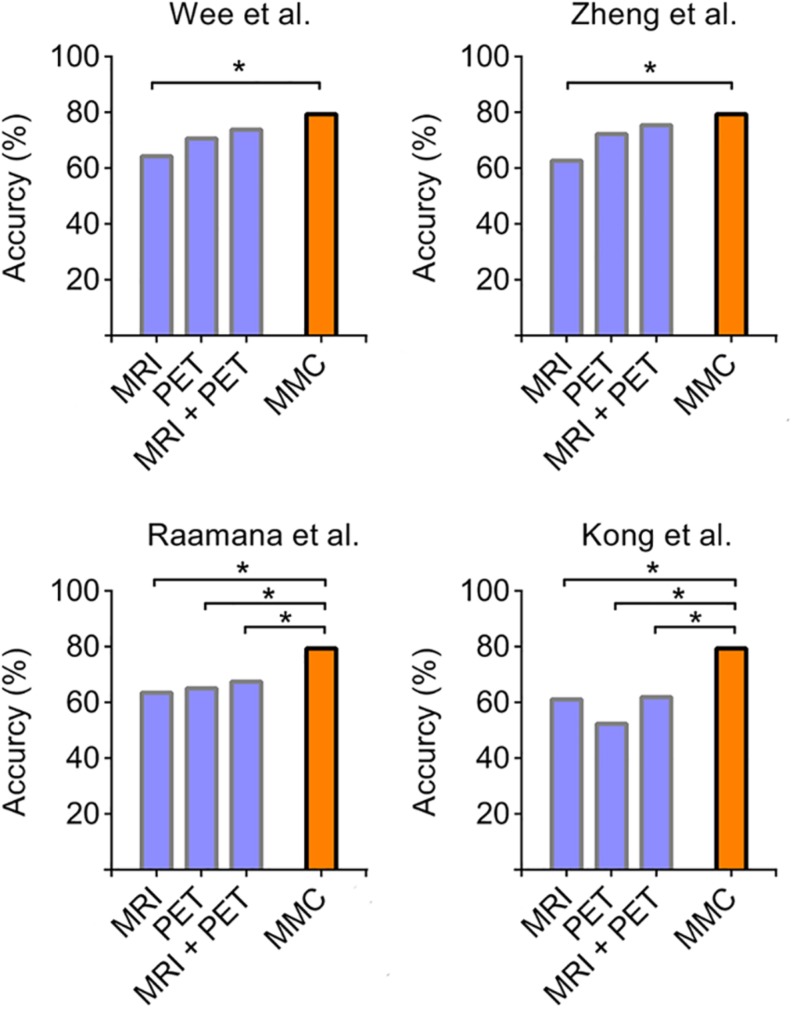
Accuracies derived from leave-one-out cross validation using different types of connectivity. Four commonly used parcellation-based connectivity extraction methods, including [opetwcite]B61,B72[clotwcite]Wee et al. (2013); Zheng et al. (2015), Raamana et al. (2015), and Kong et al. (2014), were applied to each imaging modality on our data, and were compared to the performance of the MMC. The asterisk indicates the differences between accuracies are statistically significant (*p* < 0.05, χ^2^ test).

We further compared the prediction performance of the MMC with other widely used connectivity extraction approaches (Wee et al., 2013; Kong et al., 2014; Raamana et al., 2015; Zheng et al., 2015) on the dataset we used here *via* the same leave-one-out cross validation process. As shown in Figure 4, the prediction performance of the MMC significantly outperformed most types of connectivity that extracted from distinct imaging modalities (χ^2^ test, *p* < 0.05), except the metabolic connectivity of Wee et al. (2013), Zheng et al. (2015). Though the combination of connectivity of the two modalities improved the prediction accuracies to 75.40%, it still did not surpass the accuracy of the MMC.

Comparisons of single-modal connectivity that were extracted following the same method we introduced and using LASSO instead of the multi-task model are shown in Table 3. These connectivities exhibited superior performances relative to the connectivity based on the paired-relationship of the same modality (see Figure 4), and achieved an accuracy of 76.19% when combined with the two modalities for classification (the average number of support vectors during leave-one-out cross validation was 87). However, it still did not exceed the performance of MMC. In addition, connectivity based on metabolic information outperformed the structural connectivity in predicting disease progression, which was in line with previous findings showing an excessive hypometabolism relative to atrophy (Chételat et al., 2008).

**TABLE 3:** Comparison of classification performances based on different regularization term.

**Regularization term**	**Modalities**	**Accuracy (%)**	**AUC**
*l*_1_-norm	MRI	69.05	0.7234
	PET	73.81	0.8497
	MRI + PET	76.19	0.8839
*l*_2,1_-norm	MRI + PET	79.37	0.8923

*AUC = area under curve.*

## Discussion

Extraction of an effective biomarker is one of the critical factors for improving the prediction accuracy of MCI-to-AD conversion. Numerous studies in the literature have indicated the superior performance of using cortico-cortical connectivity relative to morphological and metabolic features when identifying patients with MCI and AD from the NCs (Wee et al., 2013; Zheng et al., 2015, 2018; Yao et al., 2016; Liu et al., 2018). However, most of these connectivities are based on a single imaging modality (e.g., MRI), which omitted the potential coupling mechanism between the information possessed by different imaging modalities, therefore, limiting the performance of categorizing MCI convertors from non-convertors. In the present study, we proposed a novel connectivity extraction approach based on multi-modal images (i.e., MRI and PET), in order to enhance the performance of early AD diagnosis. The satisfactory performance achieved by the MMC suggested the effectiveness and feasibility of the multi-modal fusion strategy in connectivity extraction, and the high AUC indicated its remarkable generalizability. Possible reasons for the advanced performance of MMC are discussed below.

Multiple imaging modalities provide a comprehensive representation of abnormal alterations in the brain. MRI and FDG-PET were suggested to represent distinct information in depicting AD-related changes in the brain, i.e., structure atrophy is significantly associated with the cognitive reserve of patients with AD, whereas the development process of the disease and its clinical severity are more related to the hypometabolism of the brain (Benvenutto et al., 2018). This is complementary information that cannot be interchanged between imaging modalities for diagnosis (Zhang et al., 2011, 2012). We speculated that the combination of multi-modal information in the connectivity extraction primarily contribute to the enhanced prediction performance. Patients with MCI and AD are accompanied with atrophy and hypometabolism of GM in various cerebral structures, such as the hippocampus, posterior cingulate, and the medial temporal cortices (Baron et al., 2001; Frisoni et al., 2002; Chételat et al., 2008; Mosconi et al., 2009). Such alterations may influence the inter-regional relationship in a complex manner, thus difficult to represent *via* a single imaging modality. The present connectivity extraction approach simultaneously combines the information from different modalities, providing a more comprehensive description of the changes in the inter-regional relationship, which therefore may be more sensitive to AD progression than connectivity based on a single imaging modality.

Auxiliary domain training increases the discriminative power of the principal patterns. The subtypes of MCI may have similar alteration modes in both morphological and metabolic domains, which limits the extraction of informative features for categorization (e.g., principal alteration patterns) (Zheng et al., 2018). Since MCI is the transition stage between NC and AD, a hypothesis arises that the brain alterations of subjects with sMCI may be more like the NCs, while patients who progress to dementia may have a similar alteration mode as the AD cohort. This hypothesis has been utilized to promote the separation of MCI convertors and non-convertors, for example, using the classifiers trained by AD and NC subjects (Fan et al., 2008; Cheng et al., 2015). In the present study, we extracted the principal patterns of each brain region from the auxiliary domain of AD and NC subjects to increase discriminating ability. The projection of subjects in the target domain, using these principal patterns, may enlarge the variance between the two MCI subtypes, which therefore contributes to the enhanced prediction performance. In addition, auxiliary domain training only needs to be performed once. When we have the PCs from the auxiliary domain, it will take a few seconds to build the MMN for a new subject.

One big challenge of the neuroimaging study is that the available datasets are usually small, especially for multi-modal longitudinal data. The small samples and the large dimension of features may give rise to a biased estimation of the model. In the present study, we applied the bagging strategy which resamples the subjects with a replacement to form a sub-dataset to the PCA process rather than training PCs on all of the subjects. One benefit of using the bagging strategy is that it could give rise to a comprehensive estimation of the principal patterns in each brain region. Thus, enhancing the generalizability of the models trained using these PCs.

Studies have suggested that the linear regression model (e.g., LASSO) may additionally take the possible effects of other brain regions into account, which could take advantage of the nature of cerebral interplay, therefore making it superior to using paired correlations (e.g., Pearson correlation) (Ryali et al., 2012; Jie et al., 2014, 2016; Yu et al., 2017; Zheng et al., 2018). Our results showed that connectivity extracted by LASSO outperformed the paired relationship and were consistent with these findings. Compared to learning each task independently, multi-task learning allows using the relationship between different tasks, leading to a better model (Argyriou et al., 2007). In the present study, multi-task sparse regression was utilized to jointly find brain regions that were both morphologically and metabolically associated with the target region. Considering the unique features of the modalities and their potential interaction, the multi-task model may better characterize the overall relationships among brain regions.

Although the MMC has significantly enhanced the prediction performance, there are some limitations that need to be addressed in the future. First, the grouping criteria may contain false-categorized cases. In the present study, we defined the sMCI as subjects who maintained MCI status for 3 years (Wee et al., 2013; Zheng et al., 2015, 2018; Tong et al., 2017), however, some sMCI subjects may convert to AD after a 3-year follow up period. Since a large number of samples in the ADNI (especially for subjects who have multi-modal images) do not have longitudinal tracking information covering a period that long, this could be an important limitation that influences the discrimination (Moscoso et al., 2019). Second, we used voxel-based morphometry (VBM) analysis to extract GM volume as the structural measurement, however, there are diversified MRI features (e.g., cortical thickness and sulcal morphology) that were reported to have significant alterations in patients with MCI and AD (Du et al., 2007; Im et al., 2008; Liu et al., 2012; Yao et al., 2012), these features will be included in our future work as a potential means to enhance the classification performance. Third, the multi-task model with *l*_2,1_-norm penalty assumed all tasks share a common set of features, but omitted the information variance conveyed by different modalities (Liu F. et al., 2014). Methods to quantify an inter-regional relationship by considering the complementary information between tasks must be developed. In addition, though we have included all MCI subjects in the ADNI database who have both MRI and PET images and met the grouping criteria, replications on large independent samples would still be beneficial to examine the generalizability and validity of the MMC. In addition, we did not got control age and gender effects before network construction because it did not significantly influence the classification performance of morphological connectivity (Zheng et al., 2018), however, another study suggested a significant impact of age on classification performance when using GM density as features (Tong et al., 2017). The influences of age and gender on classification performance will be investigated in future work.

## Conclusion

In conclusion, the connectivity extracted using multi-modal measures possess important information for the categorization of MCI-convertors and non-convertors. The classification performance achieved by MMC outperformed both structural and metabolic features, as well as connectivity extracted using other common approaches. These results suggested the effectiveness of MMC in early AD diagnosis, with potential clinical implications for the auto-diagnosis of neuropsychiatric disorders.

## Data Availability Statement

Publicly available datasets were analyzed in this study. This data can be found here: http://adni.loni.usc.edu.

## Ethics Statement

The studies involving human participants were reviewed and approved by the Alzheimer’s Disease Neuroimaging Initiative. The patients/participants provided their written informed consent to participate in this study.

## Author Contributions

WZ, ZY, and BH contributed to the methodology of this study. WZ and YL processed the imaging data. WZ performed all analyses with the preprocessed data. WZ, YZ, and DW interpreted the results and contributed to writing the manuscript draft. All authors reviewed the manuscript.

## Conflict of Interest

The authors declare that the research was conducted in the absence of any commercial or financial relationships that could be construed as a potential conflict of interest.

## Footnotes

1 adni.loni.usc.edu

2 http://adni.loni.usc.edu/methods/mri-tool/mri-analysis/#mri-pre-processing-container

3 http://adni.loni.usc.edu/methods/pet-analysis-method/pet-analysis/#pet-pre-processing-container

4 www.neuro.uni-jena.de/cat12/CAT12-Manual.pdf
